# Proteomic profiling of extracellular vesicles in synovial fluid and plasma from Oligoarticular Juvenile Idiopathic Arthritis patients reveals novel immunopathogenic biomarkers

**DOI:** 10.3389/fimmu.2023.1134747

**Published:** 2023-04-27

**Authors:** Federica Raggi, Martina Bartolucci, Davide Cangelosi, Chiara Rossi, Simone Pelassa, Chiara Trincianti, Andrea Petretto, Giovanni Filocamo, Adele Civino, Alessandra Eva, Angelo Ravelli, Alessandro Consolaro, Maria Carla Bosco

**Affiliations:** ^1^ Laboratory of Molecular Biology, Istituto di Ricovero e Cura a Carattere Scientifico (IRCCS) Istituto Giannina Gaslini, Genova, Italy; ^2^ Unit of Autoinflammatory Diseases and Immunodeficiences, Pediatric Rheumatology Clinic, Istituto di Ricovero e Cura a Carattere Scientifico (IRCCS) Istituto Giannina Gaslini, Genova, Italy; ^3^ Core Facilities, Clinical Proteomics and Metabolomics, Istituto di Ricovero e Cura a Carattere Scientifico (IRCCS) Istituto Giannina Gaslini, Genova, Italy; ^4^ Clinical Bioinformatics Unit, Istituto di Ricovero e Cura a Carattere Scientifico (IRCCS) Istituto Giannina Gaslini, Genova, Italy; ^5^ Department of Neurosciences, Rehabilitation, Ophthalmology, Genetics and Maternal-Infantile Sciences (DiNOGMI), University of Genova, Genova, Italy; ^6^ Division of Pediatric Immunology and Rheumatology, Fondazione Istituto di Ricovero e Cura a Carattere Scientifico (IRCCS) Cà Granda Ospedale Maggiore Policlinico, Milano, Italy; ^7^ Pediatric Rheumatology and Immunology, Ospedale “Vito Fazzi”, Lecce, Italy; ^8^ Scientific Direction, Istituto di Ricovero e Cura a Carattere Scientifico (IRCCS) Istituto Giannina Gaslini, Genova, Italy; ^9^ Pediatric Rheumatology Clinic, Istituto di Ricovero e Cura a Carattere Scientifico (IRCCS) Istituto Giannina Gaslini, Genova, Italy

**Keywords:** oligoarticular juvenile idiopathic arthritis, proteomics, extracellular vesicles, biomarkers, inflammatory and immune processes

## Abstract

**Introduction:**

New early low-invasive biomarkers are demanded for the management of Oligoarticular Juvenile Idiopathic Arthritis (OJIA), the most common chronic pediatric rheumatic disease in Western countries and a leading cause of disability. A deeper understanding of the molecular basis of OJIA pathophysiology is essential for identifying new biomarkers for earlier disease diagnosis and patient stratification and to guide targeted therapeutic intervention. Proteomic profiling of extracellular vesicles (EVs) released in biological fluids has recently emerged as a minimally invasive approach to elucidate adult arthritis pathogenic mechanisms and identify new biomarkers. However, EV-prot expression and potential as biomarkers in OJIA have not been explored. This study represents the first detailed longitudinal characterization of the EV-proteome in OJIA patients.

**Methods:**

Fourty-five OJIA patients were recruited at disease onset and followed up for 24 months, and protein expression profiling was carried out by liquid chromatography-tandem mass spectrometry in EVs isolated from plasma (PL) and synovial fluid (SF) samples.

**Results:**

We first compared the EV-proteome of SF vs paired PL and identified a panel of EV-prots whose expression was significantly deregulated in SF. Interaction network and GO enrichment analyses performed on deregulated EV-prots through STRING database and ShinyGO webserver revealed enrichment in processes related to cartilage/bone metabolism and inflammation, suggesting their role in OJIA pathogenesis and potential value as early molecular indicators of OJIA development. Comparative analysis of the EV-proteome in PL and SF from OJIA patients vs PL from age/gender-matched control children was then carried out. We detected altered expression of a panel of EV-prots able to differentiate new-onset OJIA patients from control children, potentially representing a disease-associated signature measurable at both the systemic and local levels with diagnostic potential. Deregulated EV-prots were significantly associated with biological processes related to innate immunity, antigen processing and presentation, and cytoskeleton organization. Finally, we ran WGCNA on the SF- and PL-derived EV-prot datasets and identified a few EV-prot modules associated with different clinical parameters stratifying OJIA patients in distinct subgroups.

**Discussion:**

These data provide novel mechanistic insights into OJIA pathophysiology and an important contribution in the search of new candidate molecular biomarkers for the disease.

## Introduction

Juvenile idiopathic arthritis (JIA) is a clinically heterogeneous group of pediatric chronic inflammatory rheumatic conditions of unknown etiology and pathophysiology with onset before 16 years of age and persistence for >6 weeks and a leading cause of acquired disability (1). According to the 2001 International League of Associations for Rheumatology (ILAR) classification criteria based on disease manifestations during the first 6 months of disease (2), the most common JIA subtype in Western countries is represented by oligoarticular JIA (OJIA) (30-57% of JIA patients) (3, 4). This condition is driven by uncontrolled inflammatory responses in genetically susceptible individuals after exposure to infective/traumatic triggers or surgery and is characterized by persistent synovitis, dysregulated angiogenesis, synovial tissue (ST) hyperplasia, and progressive cartilage and bone erosion, which may lead to structural joint damage and functional impairment (1, 5–7). Arthritis arises in four or fewer joints and is characterized by early onset, female predominance, asymmetry, high frequency of anti-nuclear antibodies (ANA), strong association with HLA-DRB1*0801, and elevated risk of chronic iridocyclitis (12-30% of cases), which is the major disease complication (3). OJIA patients show considerable heterogeneity in clinical course and therapeutic outcome (8). Although the majority maintains an oligoarticular phenotype (≤4 joints), which is more benign and likely to achieve remission in response to first-line treatment (non-steroidal anti-inflammatory drugs or intra-articular glucocorticoids), a relevant percentage of them (21-39.5%) presents a polyarticular (extended) course of arthritis (involvement of ≥5 joints), which is often erosive and treatment refractory requiring more aggressive therapies (9). Despite significant advances in OJIA treatment have been obtained in the last decades due to the availability of new therapeutic agents and the possibility to perform controlled clinical trials (1), up to 40% OJIA patients do not respond adequately to therapies or fail to achieve sustained drug-free clinical remission, showing disease relapse in treated joints and/or progressive spread to other joints by one-two years after onset (3, 9, 10). Early diagnosis and prediction of disease course/outcome and iridocyclitis development in individual patients in the early stages are critical to tailor therapeutic intervention but are hindered by the lack of validated biomarkers (11–13). To date, only a few molecular, immune, and clinical markers have, in fact, been proposed (9, 14–17). Therefore, a great deal of efforts is currently being expended in the search of new biomarkers readily measurable in patient-derived material at an early stage of the disease and in a minimally invasive way (12, 18, 19). A deeper understanding of the molecular regulatory mechanisms underlying OJIA pathophysiology is critical for the discovery of candidate biomarkers and potential novel therapeutic targets.

In the last years, extracellular vesicles (EVs), a heterogeneous group of lipid bilayer membrane-delimited nanoparticles actively secreted by most cell types into biological fluids and acting as important mediators of intercellular communication by virtue of their cargo of bioactive molecules of cellular origin (nucleic acids, proteins, lipids), have attracted increasing interest as a source of biomarkers (20). EVs have been traditionally classified into two major subgroups based on differences in biogenesis and size, namely exosomes (30-150 nm in diameter), the smallest and the most well-studied class of EVs, which originate by fusion of endosomal multivesicular bodies with the plasma membrane, and microvesicles (100-1000 nm in diameter), formed by outward plasma membrane budding, although overlap between these two types of vesicles was largely reported (21, 22). EVs exert key functions in various physiological processes including immune surveillance, cell proliferation, differentiation, and apoptosis (22–25). However, they have also been shown to play crucial roles in the pathogenesis of many diseases (25, 26), including those caused by dysfunctions of the immune system (27–30). Altered EV-protein (EV-prot) profiles were found closely linked to the development and progression of various chronic inflammatory and autoimmune disorders (e.g. rheumatoid arthritis, RA) (30, 31), and EV proteomic profiling has recently emerged as a promising approach for the characterization of the molecular bases of disease pathogenesis and progression (29, 30, 32, 33), contributing to the discovery of new “omics” biomarkers (34–36). Although a few studies have used a proteomics approach to interrogate biologic fluids of children affected by different types of JIA (16, 37–40), EV-prot expression and potential as biomarker in OJIA have not been investigated.

Because joints are the main targets of clinical manifestations in OJIA, it is probable that the most relevant biomarkers of disease development and potential therapeutic targets would be localized within affected joints (41, 42). Synovial fluid (SF) is a protein-rich fluid produced into the joint cavity by cells of the synovial membrane in strict contact to tissues primarily altered during articular disease, thus reflecting the biochemical milieu of the joint and offering a direct measure of its pathologic state (43). Previous studies in patients affected by RA have shown that EVs are actively released into the SF by local and infiltrating immune cells within synovial joints and contribute to the perpetuation of joint inflammation, synovial cell proliferation, and cartilage degradation (28, 29, 41, 44). Characterization of the EV-prot pattern in SF from OJIA patients early at disease presentation may, thus, provide new insights into the molecular mechanisms underlying articular pathology and allow to derive novel early biomarkers with diagnostic or prognostic potential.

The present study was aimed at characterizing the EV-prot expression profile in SF and plasma (PL) of children newly diagnosed with OJIA. Our results define specific EV-prot expression patterns in SF at disease onset, which might contribute to the regulation of important biological processes implicated in disease development. In addition, we identify an EV-prot signature in PL samples differentiating new-onset OJIA patients from control children and, thus, representing early putative diagnostic biomarkers. Finally, we identify distinct clusters of EV-prots highly associated with patient clinical parameters differentiating subgroups of patients with different disease course at 2 years of follow-up, suggesting their potential as early indicators of disease outcome.

## Materials and methods

### Study population

Forty five patients who met the 2001 International League for Associations of Rheumatology (ILAR) classification criteria for OJIA (i.e. disease involvement of ≤4 joints in the first 6 months of disease) (2) undergoing arthrocentesis at the Pediatric Rheumatology, IRCCS Gaslini Institute, Genova, SC Pediatric Immuno Rheumatology, IRCCS Ca’ Granda Maggiore Hospital, Milano, and Pediatric Rheumatology and Immunology, Hospital “Vito Fazzi”, Lecce, as part of clinical care were enrolled consecutively in the study from October 2018 through June 2020 and followed up every 3 months for 2 years after disease diagnosis (June 2022). All patients had clinically active disease, with joint effusion, swelling, pain, and stiffness at the time of sampling, and onset of symptoms for no more than 6 months before enrollment. The number of active joints was determined by standard clinical evaluation followed by ultrasound or MRI evaluation. Arthrocentesis was performed at the time of disease diagnosis under local anesthesia or, in case of younger patients or multiple joints, under general anesthesia. Various clinical and laboratory parameters were measured. A previous or current treatment with anti-inflammatory drugs was considered as an exclusion criterion. Patients underwent different therapeutic regimens after diagnosis during the follow-up period. Disease relapse (referred to as occurrence of new flares within the onset joints or other joints), polyarticular extension, and iridocyclitis development were determined prospectively during the longitudinal follow up evaluation. No attempt was made to segregate patients on the basis of disease duration, number of active joints involved at disease onset, or therapeutic regimen during follow-up. Twenty-four age- and gender-matched children undergoing minor orthopedic procedures at the Gaslini Institute were enrolled as a control group. Detailed clinical and laboratory examination of control subjects was carried out to rule out infections, inflammatory, and chronic diseases. The main demographic, clinical, laboratory, and therapeutic characteristics of the study cohorts are reported in **Table 1**. The protocol of the study was reviewed and approved by the Ethics Committee of the Region Liguria (Approval 165/2018), the Ethics Committee Milano Area 2 (Approval 639/2019), and the Ethics Committee ASL Lecce (Approval N° 36/2019), and the procedures were carried out according to the approved guidelines and in adherence to the general ethical principles set forth in the Declaration of Helsinki. Written informed consent to participate in the study was obtained from the parents or the patient’s legal guardian prior to sample collection.

**Table 1:** Demographic, clinical, laboratory, and therapeutic features of OJIA patients at disease onset and follow up^a^.

	Patients (n=45)	CTR (n=24)
**Female^b^**	25 (55.5)	13 (54.1)
**Age at onset (yrs)^c^**	5.25 (0.12-14.21)	7.21 (0.49-13.97)
**CRP (mg/dL)^c, £^**	0.8 (0.46-7)	<0.46
**ESR (mm/h) ^c, $^**	25 (3-77)	-
**JADAS 10 ^c,&^**	11.5 (2-18)	-
**Patients with ANA positivity^b^**	19 (42.2)	-
**No. of active joints (at sampling)^b^**		
One	23 (51.1)	-
Two	16 (35.5)	-
Three	2 (4.4)	-
Four	4 (8.8)	-
**Type of involved joints^b^**		
Knee	43 (95.5)	-
Elbow	2 (4.4)	-
Wrist	1 (2.2)	-
Ankle	3 (6.6)	-
Metacarpophalangeal	2 (4.4)	-
Metatarsophalangeal	1 (2.2)	-
Proximal Interphalangeal	2 (4.4)	-
Tibio tarsal	3 (6.6)	-
Subtalar	2 (4.4)	-
Talonavicular	3 (6.6)	-
Interapophyseal	1 (2.2)	-
**Relapse (within 2 yrs)^b^**	28 (62.2)	-
**Polyarticular extension (within 2 yrs)^b^**	11 (24.4)	-
**Patients with iridocyclitis^b^**	7 (15.5)	-
**Drug therapies (after onset)^b,d^**		
IAS	45 (100)	-
NSAIDs	2 (4.4)	-
MTX	10 (22.2)	-
**Drug therapies (after 1st recurrence)^b,e^**		
None	17 (37.7)	-
IAS	24 (53.3)	-
MTX	16 (35.5)	-
Steroid	4 (8.8)	-

^a^The table reports the main demographic, clinical, laboratory, and therapeutic parameters of the patients enrolled in the study. Disease activity was defined by the presence of joint swelling or limitation of movement with either pain on movement or tenderness. Patients were followed up for 2 years after disease onset.

^b^Results are expressed as number of patients (percentage in parenthesis).

^c^Results are expressed as mean (range in parenthesis).

^d^Drug therapies administered after disease initial presentation.

^e^Drug therapies administered after the first disease flare.

^£^Available for 44 patients; ^$^ available for 41 patients; ^&^ available for 10 patients.

^§^None: not available.

ESR, erithrocyte sedimentation rate; CRP, C-reactive protein; ANA, anti-nuclear antibodies; IAS, Intra-articular steroid; NSAIDs, Non-steroidal anti-inflammatory drugs; MTX, Methotrexate.

### Sample collection

**Figure 1:**
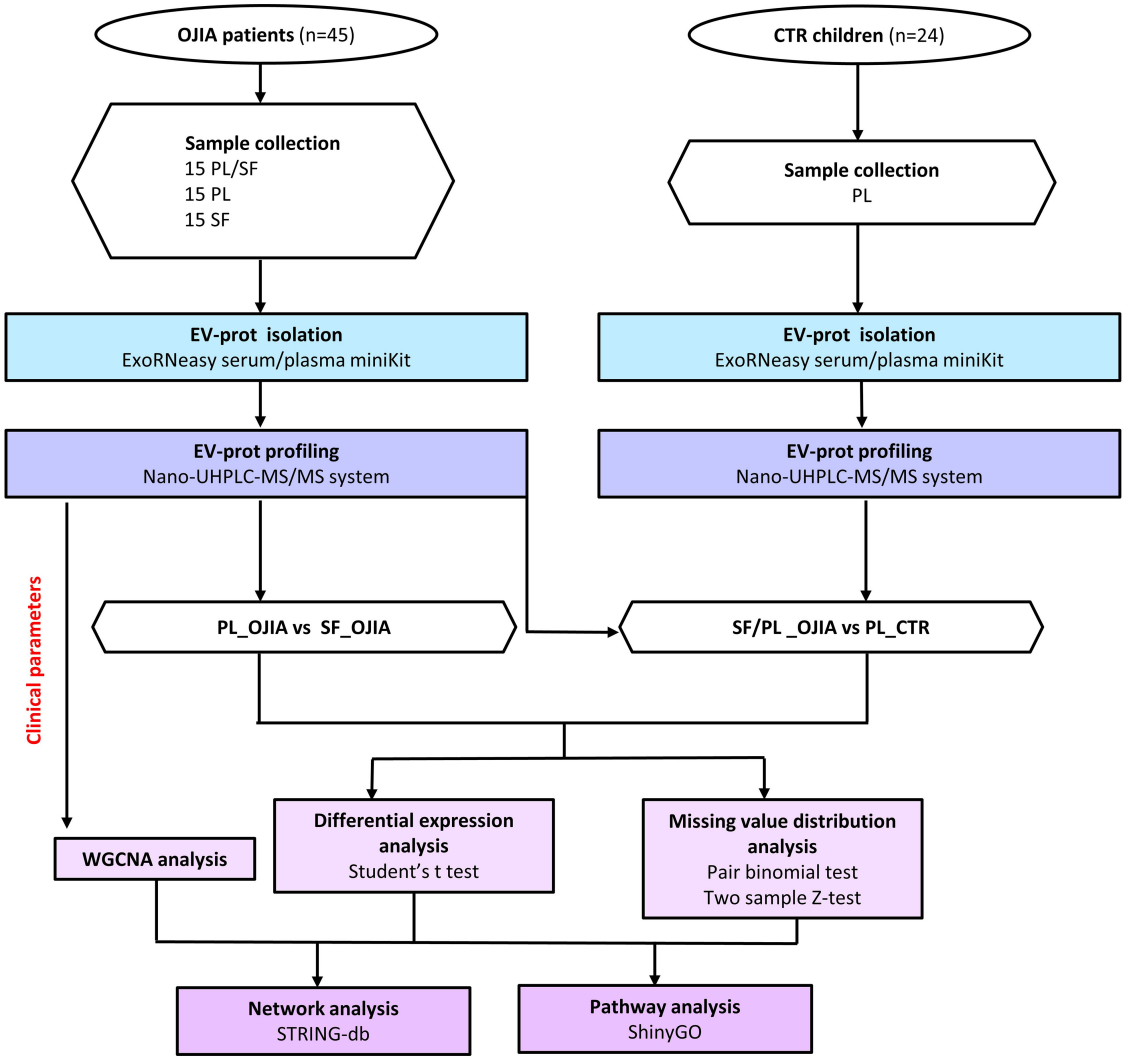
Flow chart of SF and PL sample processing and EV-prot isolation/analysis in OJIA patients and control children. SF and PL samples from forty-five newly diagnosed OJIA patients with clinically active disease and PL from twenty-four control children were collected, and EVs were purified using the membrane affinity spin column, exoRNeasy Serum/Plasma kit. EVs were lysed with RIPA buffer, and EV lysates were processed according to the in-StageTip procedure. EV-prot expression profile was analyzed by liquid chromatography-tandem mass spectrometry (LC-MS/MS). EV-prot expression was then compared among the different groups of specimens. Differential expression analysis was carried out by paired or two-test Student’s t test method; analysis of distribution of missing values was assessed by a matched pair binomial test or two sample Z-test for proportions. Protein-protein interaction network analysis were carried out on selected statistically significantly differentially expressed EV-prots using STRINGApp and functional annotation. Pathway analysis was performed using the ShinyGO web server. Weighted gene co-expression network analysis (WGCNA) was performed on the SF_OJIA and PL_OJIA EV-prot datasets based on selected patient clinical parameters achieved by 2 years of follow-up.

Synovial fluid (SF) aspirates were collected from affected joints of thirty OJIA patients (SF_OJIA) at the time of arthrocentesis under vacuum into tubes containing EDTA, as described in Ref (45). Peripheral blood (PB) samples from thirty OJIA patients (PL-OJIA) and twenty-four control children (PL_CTR) were obtained by venipuncture and collected in EDTA tubes. Among them, fifteen PB and SF samples were derived from the same OJIA patients (**Figure 1**). Specimens were centrifuged at 500 x g for 10 minutes at room temperature (RT) to obtain cell-free SF and PL and stored at -80°C until use.

### EV and EV-prot isolation

EV isolation from 500 μl of SF and PL samples was performed using the exoRNeasy Serum/Plasma Midi kit (Qiagen Italia, Milano, Italy), that uses membrane affinity spin columns to efficiently capture intact EVs from small sample volumes, according to the manufacturer instructions, as described (45). Cell-free SF samples were either treated with 2U/ml Hyaluronidase (HYase) (Sigma, Merck Life Science, Milano, Italy) for 30 minutes at 37°C to remove contaminating hyaluronan extracellular matrix (ECM) components. Samples were firstly centrifuged at 500 x g for 10 minutes at RT and then at 16,000 × g for 15 minutes at RT to eliminate cellular debris. PL samples, not undergoing HYase treatment, were directly centrifuged at 16,000 × g for 15 minutes. Supernatants were mixed with one volume of XBP binding buffer and loaded onto the exoEasy spin column to bind EVs to the membrane. After centrifugation at 500 x g for 1 minute at RT, the flow-through was discarded and the column was washed with 2 ml of XWP Washing Buffer and spun at 2,500 ×g for 5 minutes at RT. This step was repeated twice to properly remove any contaminating agent. Then, the column was washed with 3.5 ml of XWP Washing Buffer and spun at 5,000 x g for 5 minutes at RT, to wash-off not specifically bound material. The spin column was transferred to a new collection tube and EVs were lysed directly in the column by addition of 100 μl of RIPA Buffer (Thermo Fisher Scientific, Milano, Italy) and incubated for 5 minutes at 4°C. The column was then centrifuged 500 × g for 5 minutes, and EV-prot lysates were collected.

### Mass spectrometry (MS)

EV lysates were processed according to the in-StageTip procedure, as described previously (46, 47). 100 ul of EV lysates in RIPA buffer were reduced and alkylated with 10 mM TCEP and 4 mM CAA in thermomixer 10 min at room temperature and at 1000 rpm. Proteins were then isolated by the PAC method (48). Briefly, protein aggregation was induced by addition of 70% CAN, and 200 μg of magnetic beads were added to capture aggregated proteins. Magnetic beads were retained by the magnet and the supernatant was removed. Beads were washed one time with 1 ml acetonitrile, followed by one wash with 1 ml 70% ethanol and one wash with 1 ml isopropanol. Washed beads were resuspended in 100 μl TRIS 25 mM pH 8, and captured proteins were digested O.N. at 37°C with 0.7 μg Trypsin and 0.3 μg LysC. Obtained peptides were desalted in Stage-Tips (49) (normalization to 7 ug peptides) and analyzed by a nano-UHPLC-MS/MS system using an Ultimate 3000 RSLC coupled to an Orbitrap Fusion Tribrid mass spectrometer (Thermo Scientific Instrument). Peptide elution was performed with an EASY spray column (75 μm x 25 cm, 2 μm particle size, Thermo Scientific) at a flow rate of 400 nl/min using a linear gradient of 7-45% solution B (80% ACN and 20% H2O, 5% DMSO, 0.1% FA) in 65 min. MS analysis was performed in DDA mode. Orbitrap detection was used for MS1 measurements at a resolving power of 120K in a range between 375 and 1500 m/z and with an AGC target of 400000, maximum injection time 50 ms. Advanced Peak Determination was enabled for MS1 measurements. MS/MS spectra were acquired in the linear ion trap (rapid scan mode) after higher-energy C-trap dissociation (HCD) at a collision energy of 28% and with an AGC target of 10000, maximum injection time 22 ms. 2 sec. cycle time was performed for data dependent MS/MS analysis, during which precursors with a charge range 2-5 were selected for activation in order of abundance. Quadrupole isolation with a 1.6 m/z isolation window was used, and dynamic exclusion was enabled for 15 s.

### Bioinformatic analysis

Protein raw data were processed with MaxQuant software (50) version 2.0.3.0. A false discovery rate (FDR) of 0.01 was set for identifying proteins, peptides, and peptide-spectrum match (PSM). For peptide identification, a minimum length of 7 amino acids was required. Andromeda engine, incorporated into MaxQuant software, was used to search MS/MS spectra against the Uniprot human database (release UP000005640_9606 July 2021). In the processing the Acetyl (Protein N-Term), Oxidation (M) and Deamidation (NQ) were selected as variable modifications and the fixed modification was Carbamidomethyl (C). Quantification intensities were calculated by the default fast MaxLFQ algorithm with the activated option ‘match between runs’. The resulting protein groups were analyzed using the Perseus software, version 1.6.15.0 (51). Contaminants and decoys were filtered out and LFQ values were subsequently log2 transformed for further statistical analysis. Proteins with at least 30% of valid values in each group were retained for the analysis prior to any relative quantification. Missing values (NA) in retained samples were imputed (52, 53) from a normal distribution using a width of 0.3 and a downshift of 1.8.

Principal Component Analysis (PCA) with default parameters was performed using Perseus algorithm to visualize correlated groups of data. The first two principal components were plotted. Volcanoplot and heatmap representation with unsupervised hierarchical clustering analysis were carried out to visualize proteomic data using the Perseus software. Overlapping and exclusive elements among the lists of EV-prots were defined by Venn diagrams using InteractiVenn web-based tool (54).

Protein-protein interaction (PPI) network analyses were carried out on selected significantly deregulated EV-prot using the Search Tool for the Retrieval of Interacting Genes (STRINGApp in Cyoscape 3.9.1) (55) to construct functional interaction networks among proteins. PPI enrichment p-value was used to assess whether proteins in a group of have more interactions among themselves than what would be expected from a random set of proteins of similar size, drawn from the genome. We set up a required minimum confidence score of 0.4 (medium confidence) and considered significant PPI enrichment p value <0.05.

Gene ontology (GO) enrichment analysis was conducted on deregulated proteins using the ShinyGO web server (version 0.76.1), a large annotation database derived from Ensembl and STRING-db (56). The human proteome served as a reference for the GO enrichment analysis. EV-prot were classified according to the GO biological process collection. An FDR < 0.05 was considered significant, and fold enrichment scores were used to assess the significance of resulting GO terms and pathways enrichment.

Weighted correlation network analysis (WGCNA) was carried out separately on SF_OJIA and PL_OJIA using a specific software R package in the Perseus platform to construct coexpression modules associated with patient clinical parameters, as described in (57, 58), after filtering of 50% of valid values in total for both PL_OJIA and SF_OJIA. The power parameter, selected by a ‘‘Soft-threshold’’ activity set on a scale-free fit index of 0.9, for PL_OJIA was 8, while for SF_OJIA was 18. Both network types were set to signed with a cor correlation function.

### Statistical analysis

The potential confounding effect of age or sex (male vs female) between CTR subjects and OJIA patients was assessed by Mann-Whitney test or Fisher’s exact test, respectively. Normality or lognormality of numerical distributions was assessed by D’agostino & Pearson test. P values lower 0.05 were considered statistically significant.

Two-sample Student’s t-test was used to identify differences in the EV-prot expression levels when the comparison involved samples derived from OJIA patients and CTR subjects (i.e., SF_OJIA vs PL_CTR and PL_OJIA vs PL_CTR). Paired Student’s t-test was applied to determine the significance of EV-prot expression differences between paired PL and SF samples, after further filtering for the minimum 30% of valid values in each of the two groups (e.g., SF_OJIA vs PL_OJIA). For both two-sample Student’s t-test and paired Student’s t-test only EV-prots with log2 fold change higher than 1 or lower than -1 were considered significantly modulated. To reduce the probability of false positive findings deriving from multiple hypothesis testing a permutation-based false discovery rate (FDR) p-value lower than 0.01 was applied.

Two sample Z-test for proportions was used to detect EV-prots whose distribution of missing values between OIJA and CTRL samples resulted statistically significantly different. Proteins with z-test scores lower than -4.0 or higher than 4.0 (p<0.00005) were considered to have statistically significant differences in the distribution of missing values. When PL and SF samples were relative to the same patients, the statistically significant difference in the distribution of missing values was assessed by a matched pair binomial test. Differences with p-value lower than 0.0001 were considered statistically significant. Study sample size (power) calculation was performed by G*Power software version 3.1 (59).

## Results

### Analysis of protein expression profiles in SF- and PL-derived EVs from new-onset OJIA patients and control subjects

Experiments were carried out to characterize the protein expression profile of EVs released into the SF and PL of new-onset OJIA patients. Forty-five OJIA patients with clinically active disease were enrolled in the study at the time of disease diagnosis and therapeutic arthrocentesis, and SF and PL samples were collected. PL samples were obtained in parallel from twenty-four age- and gender-matched CTR children. The demographic (age and sex) and main clinical features of patients and control subjects, various laboratory parameters and known markers of disease activity such as erythrocyte sedimentation rate (ESR), C-reactive protein (CRP), and anti-nuclear antibodies (ANA), the number and type of active joints at onset, therapeutic regimens administered after diagnosis, disease oligoarticular or polyarticular course, and iridocyclitis development within a 2 years follow-up period are reported in **Table 1**. The 2 years follow up was set up because polyarticular extension in OJIA occurs more frequently within this time after initial disease presentation (9). Mean patient age at onset was 5.2 years and female/male ratio was 25 (55,6%)/20 (44,4%), whereas mean control subject age was 7.2 years and female/male ratio was 13 (54.2%)/11 (45.8%). Association between age or sex (male vs female) and subject group (OJIA vs CTR) was investigated to estimate whether demographic features could represent potential confounding factors in the present study. Mann-Whitney test and Fischer’s exact test did not show any significant association between the case and control groups analyzed with regard to age or sex, respectively (p>0.05), thereby excluding a potential confounding effect of these characteristics. Positivity for ANA was observed in nineteen patients (42%). Twenty-three patients (51%) had involvement of 1 joint, whereas sixteen patients (35.5%) had 2 affected joints, and only six patients (13%) had arthritis in more than 2 joints. The most frequently involved joint was the knee (fourty-three patients, 95.5%), whereas all other joints were affected in three or fewer patients (< 7%). The most common therapeutic intervention after diagnosis was intra-articular glucocorticoids (all patients), whereas only a subset of ten patients was given methotrexate (MTX) (22.2%) and two received NSAID (4.4%). Treatment with MTX or other anti-inflammatory drugs was often administered to patients showing disease relapse. At the end of the 2 years follow up, twenty-eight patients (62.2%) had experienced new disease flares, eleven (24.4%) developed polyarticular extension, while thirty-four (75.6%) maintained an oligoarthritis phenotype, and seven patients (15,6%) developed iridocyclitis. The general features of the cohort of patients enrolled in the present work are similar to those described in other previous studies (9). We performed an *a priori* sample size calculation with G*Power software (effect size d=1.0, α = 0.05, 1-β = 0.95, standard deviation=1.0) to assess a fold change difference of at least 2.0 between OJIA patients and CTR groups. The total sample size resulted of 54 subjects, which is below the total number of subjects (patients and CTR) enrolled in the study, confirming that the study contains enough power to make a reasonable conclusion.

**Figure 2:**
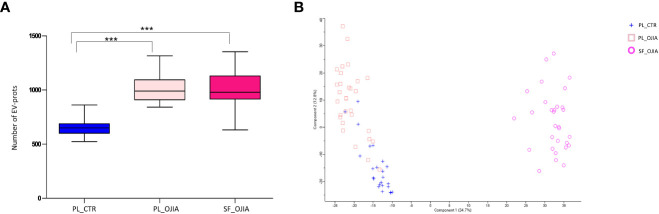
Comparative analysis of EV-prot expression profiles among SF_OJIA, PL_OJIA, and PL_CTR specimens. EV-prots were profiled in SF and PL from new-onset OJIA patients and PL from control children by LC-MS/MS. **(A)** The box plot indicates the average number of EV-prots detectable in the different biological groups. Boxes comprise the values falling between the 25th and 75th percentiles, median values are represented by horizontal lines, and the highest and lowest values for each group are represented by whiskers (lines that extend from the boxes). Statistical analysis using unpaired Student’s t-test was performed. P value of PL_CTR relative to OJIA PL and SF: ***p<001. **(B)** Principal Component Analysis (PCA) shows separation among different biological groups on the basis of the EV-prots expression profiles. The percentage of the total variation accounted for the first and second components is shown on the x and y axes, respectively. Blue crosses, pink squares, and fuchsia circles refer respectively to PL_CTR, PL_OJIA and SF_OJIA. PL and SF groups form two clusters on Principal Component 1 (PC1) showing high differences at the proteome level. OJIA_PL and PL_CTR partially cluster on PC2.

EVs were isolated from collected samples according to our recently set up experimental protocol (45). The characteristics of EVs isolated using this procedure in terms of size, morphology, and marker expression were reported previously (45). The number of EVs isolated from SF and PL ranged between 5.14x10^10^-1.22x10^11^ and 7.06x10^10^-1.48x10^11^ particles/ml, respectively, as quantified by NTA. EV protein cargo was profiled using high-resolution mass spectrometry coupled with liquid chromatography (LC-MS/MS). A flowchart summarizing the main steps of sample processing and data analysis is depicted in **Figure 1**. A total of 2444 proteins were identified. Proteins resulting after filtration of the most common contaminants (e.g., albumin, cytoskeletal keratins, collagen-alpha-1, tropomyosin-beta, thrombospondin-1) were 2282. Greater protein abundance was detectable in both SF (average number 1015 ± 31.9) and PL specimens (average number 1013 ± 23.4) from OJIA patients respect to PL from CTR (average number 657 ± 17.7) (**Figure 2A**).

EV-prot expression patterns were then determined in the different groups of specimens. We carried out both EV-prot differential expression and missing value distribution analysis. Differential expression was evaluated by comparing the levels of common EV-prots among groups by the Student’s t test method, applying a filter based on NAs. Only EV-prots with at least 30% of valid values in each group were retained for imputation and analysis, whereas the rest was filtered out (as detailed in the Materials and Methods). The filtration step reduced the total number of proteins to 594. As shown by the PCA reported in **Figure 2B**, SF_OJIA, PL_OJIA, and PL_CTR samples could be clearly clustered into three well-defined groups based on their EV-prot expression profiles, with substantial homogeneity among samples belonging to the same group. Analysis of the distribution of missing values was investigated on all the 2282 EV-prots to identify those exclusively expressed in each sample group. A matched pair binomial test was applied to analyze paired PL_OJIA vs SF_OJIA samples, whereas the two sample Z-test for proportions was applied to analyze unpaired OJIA and CTR samples.

### EV-prot expression patterns differ between paired SF and PL samples from new-onset OJIA patients

To identify EV-prots potentially implicated in OJIA joint pathogenesis, we conducted a comparative analysis of the proteome of EVs isolated from SF_OJIA respect to that of paired PL_OJIA samples from newly-diagnosed OJIA patients. Statistically significant differences in EV-prot expression levels between groups were visualized by volcano plot and heat map representation (**Supplementary Figure 1**). Volcano plot revealed a clear separation between SF and PL samples on the basis of the EV-prot expression profiles (Panel A). Unsupervised hierarchical clustering analysis confirmed the presence of distinct EV-prot patterns in paired SF and PL samples, with substantial homogeneity among samples belonging to the same group (Panel B). To define EV-prots that mostly contributed to the observed differences, we performed a differential expression analysis of the proteomics datasets and identified a total of 235 statistically significantly modulated EV-prots (FDR<0.01) between the two groups of specimens, of which 110 and 125 were specifically up- and downregulated in SF *vs* paired PL, respectively. The list of modulated EV-prots is reported in **Supplementary Table 1**. These results indicate that the protein expression profile of EVs isolated from the joints of new-onset OJIA patients strongly differs from the systemic profile.

**Figure 3:**
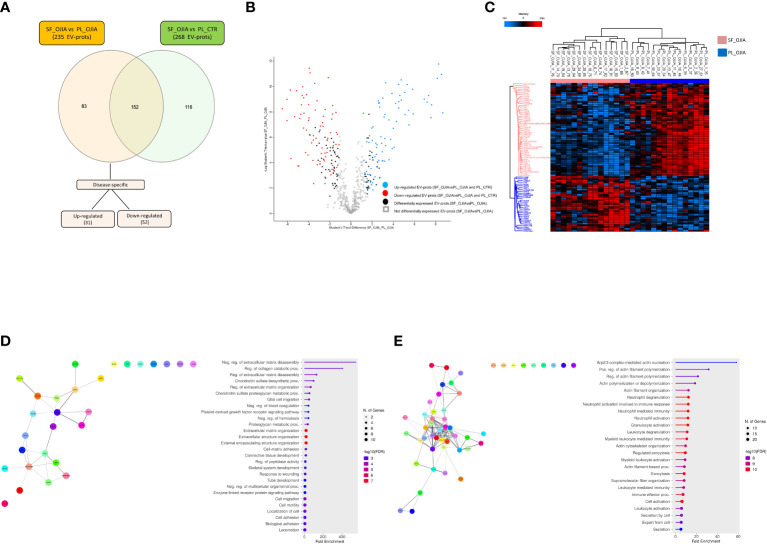
Specific EV-prot expression pattern differences between paired SF and PL specimens from new-onset OJIA patients. EV-prots isolated from paired SF and PL samples of new-onset OJIA patients and PL of CTR children were profiled, and those exclusively differentially expressed in the comparison SF_OJIA vs PL_OJIA respect to SF_OJIA vs PL_CTR were identified and subjected to network and functional enrichment analyses. **(A)** Venn diagram shows the number of common and exclusive differentially expressed EV-prots detectable between the comparison of SF-OJIA vs PL-OJIA and SF-OJIA vs PL-CTR. Each comparison is depicted by a distinct color. Up- and downregulated exclusively differentially expressed EV-prots are indicated. The significance of the overlapping was estimated with hypergeometric statistics. **(B)** Volcano plot shows the changes in protein expression levels between the two comparisons. Significance (–log10 of p value) is plotted on the y axis versus fold-change (log2) on the x axis. On the left are shown EV-prots significantly downregulated and on the right those significantly up-regulated in OJIA_SF samples with respect to PL_OJIA. **(C)** Heat-map representation shows unsupervised hierarchical clustering analysis of EV-prots specifically differentially expressed in paired SF_OJIA vs PL_OJIA groups of samples. EV-prot expression levels were z-scored and log2 transformed and are indicated by a two-color scale ranging from blue (lowest values) to red (highest values) reported in the horizontal bar at the top of the figure. Each column corresponds to a sample (indicated on the top side) and each row represents a differentially expressed protein derived from the comparison. The pink and blue bars above the columns distinguish the SF and PL groups. Dendrogram reporting the results of the unsupervised hierarchical clustering is displayed at the top of the plot. Differences in EV-prot expression levels were considered statistically significant when s0 = 1 and FDR = 0.01. The optimal association between two main clusters of EV-prots (indicated by the pink and blue bar on the left side) identified by the unsupervised hierarchical method and the SF and PL groups of samples is shown **(D, E)** Network and pathway enrichment analyses were carried out on the subset of EV-prots exclusively up- **(D)** and down- **(E)** regulated in paired SF_OJIA vs PL_OJIA samples. Functional interaction networks among EV-prots were built using STRINGApp and are displayed graphically as nodes (EV-prots) and edges (predicted protein–protein interactions). Colored nodes indicate query EV-prots. Filled nodes indicate some 3D structure known or predicted. Line thickness indicates the strength of data support. Medium confidence of 0.4 was set up as the minimum required confidence score, and a PPI enrichment FDR value < 0.05 was considered significant. Functional enrichment analysis for GO biological processes were conducted by ShinyGO, and enriched pathways were considered significant if FDR value ≤0.05. The graph shows the top enriched terms. EV-prots might appear in more than one term depending on their function. The GO term name is reported on the y-axis, fold enrichment for each term is indicated on the x-axis. GO terms are listed by decreasing fold enrichment value.

Because the observed differences in EV-prot expression levels could be due at least in part to the diverse nature of the fluid analyzed (SF vs PL) and, thus, not be specific to the disease state, we compared the 235 EV-prots identified in SF_OJIA vs PL_OJIA with those resulting from the comparison between SF_OJIA and PL_CTR samples and filtered out proteins commonly differentially expressed in the two comparisons and thus potentially related to the specific fluid characteristics. Volcano plot (**Supplementary Figure 1C**) and heat map (**Supplementary Figure 1D**) clearly clustered SF_OJIA and PL_CTR specimens into two well-defined groups based on their EV-prot expression levels, with a total of 268 proteins (130 up- and 138 down-regulated) identified as significantly differentially expressed (FDR < 0.01) (**Supplementary Table 2**). Common and exclusive differentially expressed proteins resulting from the comparison of the two datasets were visualized by the Venn diagram and the Vulcano plot depicted in **Figures 3A, B**. 152 EV-prots were commonly modulated in paired SF *vs* PL from OJIA patients and SF_OJIA vs PL_CTR (indicated by red and blue circles in the Volcano plot), whereas a restricted subset of 83 EV-prots was specifically modulated upon comparison of SF_and PL_OJIA samples (black circles in the Vulcanoplot), probably representing protein specifically related to the disease. Among them, 31 were up-regulated and 52 down-regulated (**Figures 3A, C**). The complete list of this panel of proteins is reported in **Table 2**. These data demonstrate the existence of a SF-associated EV-prot signature with potential value as an early molecular indicator of the joint pathologic state.

**Table 2:** Relative expression of EV-prots specifically up and down-regulated in SF_OJIA vs PL_OJIA^a^.

Protein IDs^b^	Protein names	Gene names^c^	Adjusted p value^d^	FC^e^
Up-regulated				
P02458	Collagen alpha-1(II) chain	COL2A1	3.57E+00	2.67
A0A0D9SEN1	Prolyl endopeptidase FAP	FAP	4.05E+00	2.52
P05109	Protein S100-A8	S100A8	3.66E+00	2.46
A0A0U1RQV3	EGF-containing fibulin-like extracellular matrix protein 1	EFEMP1	2.26E+00	2.38
P05154	Plasma serine protease inhibitor	SERPINA5	2.68E+00	2.15
Q12797	Aspartyl/asparaginyl beta-hydroxylase	ASPH	4.20E+00	2.08
Q6EMK4	Vasorin	VASN	4.40E+00	2.04
Q6UVK1	Chondroitin sulfate proteoglycan 4	CSPG4	4.82E+00	1.96
Q12841-2	Follistatin-related protein 1	FSTL1	3.01E+00	1.85
A2NJV5		IGKV A18	2.55E+00	1.84
P01743	Ig heavy chain V-I region HG3		4.90E+00	1.80
P13611	Versican core protein	VCAN	4.56E+00	1.72
P08571	Monocyte differentiation antigen CD14	CD14	7.83E+00	1.70
A0A075B6I0		IGLV8-61	3.70E+00	1.66
Q7LGC8	Carbohydrate sulfotransferase 3	CHST3	2.58E+00	1.65
A0A024R6I7		SERPINA1	2.14E+00	1.65
Q07954	Prolow-density lipoprotein receptor-related protein 1	LRP1	2.01E+00	1.64
Q15582	Transforming growth factor-beta-induced protein ig-h3	TGFBI	5.95E+00	1.64
Q9NT62	Ubiquitin-like-conjugating enzyme ATG3	ATG3	2.59E+00	1.63
Q14112-2	Nidogen-2	NID2	2.05E+00	1.57
P05090	Apolipoprotein D	APOD	1.86E+00	1.57
Q9BYJ0	Fibroblast growth factor-binding protein 2	FGFBP2	1.53E+00	1.56
B1AMW1	Lymphocyte function-associated antigen 3	CD58	2.47E+00	1.52
P04004	Vitronectin	VTN	5.94E+00	1.51
P01034	Cystatin-C	CST3	2.02E+00	1.48
Q9HDC9	Adipocyte plasma membrane-associated protein	APMAP	4.04E+00	1.42
P13942-6	Collagen alpha-2(XI) chain	COL11A2	2.48E+00	1.41
Q96IY4	Carboxypeptidase B2	CPB2	2.90E+00	1.41
P09603	Macrophage colony-stimulating factor 1	CSF1	3.09E+00	1.33
Q5VY30	Retinol-binding protein 4	RBP4	3.17E+00	1.33
C9JA05	Immunoglobulin J chain	JCHAIN	2.92E+00	1.33
Down-regulated				
P30101	Protein disulfide-isomerase A3	PDIA3	4.40E+00	-1.15
F8WCF6	Actin-related protein 2/3 complex subunit 4	ARPC4-TTLL3	4.51E+00	-1.24
O15511	Actin-related protein 2/3 complex subunit 5	ARPC5	3.02E+00	-1.24
P23528	Cofilin-1	CFL1	5.13E+00	-1.26
P0DJI8	Serum amyloid A-1 protein	SAA1	2.65E+00	-1.29
Q32Q12	Nucleoside diphosphate kinase	NME1-NME2	2.93E+00	-1.33
A0A087WTK0	Protein-tyrosine-phosphatase	PTPRJ	4.48E+00	-1.35
Q07960	Rho GTPase-activating protein 1	ARHGAP1	2.21E+00	-1.37
O15144	Actin-related protein 2/3 complex subunit 2	ARPC2	3.11E+00	-1.38
Q13561	Dynactin subunit 2	DCTN2	2.10E+00	-1.46
P06753-2		TPM3	4.09E+00	-1.48
P61026	Ras-related protein Rab-10	RAB10	2.33E+00	-1.49
Q14344	Guanine nucleotide-binding protein subunit alpha-13	GNA13	2.37E+00	-1.52
P68133	Actin, alpha skeletal muscle	ACTA1	4.80E+00	-1.54
Q9H0U4	Ras-related protein Rab-1B	RAB1B	4.86E+00	-1.55
O15400-2	Syntaxin-7	STX7	1.98E+00	-1.56
P28070	Proteasome subunit beta type-4	PSMB4	3.35E+00	-1.56
Q15907	Ras-related protein Rab-11B	RAB11B	3.57E+00	-1.57
P78417	Glutathione S-transferase omega-1	GSTO1	3.65E+00	-1.58
P07737	Profilin-1	PFN1	5.62E+00	-1.63
O14672	Disintegrin and metalloproteinase domain-containing protein 10	ADAM10	3.15E+00	-1.67
P37802	Transgelin-2	TAGLN2	4.94E+00	-1.67
P25786	Proteasome subunit alpha type-1	PSMA1	2.23E+00	-1.69
Q01518	Adenylyl cyclase-associated protein 1	CAP1	5.16E+00	-1.72
K4DIA7	Tetraspanin	CD151	2.36E+00	-1.74
O75563	Src kinase-associated phosphoprotein 2	SKAP2	4.19E+00	-1.80
Q04917	14-3-3 protein eta	YWHAH	5.91E+00	-1.83
O15143	Actin-related protein 2/3 complex subunit 1B	ARPC1B	3.94E+00	-1.84
E9PP21	Cysteine and glycine-rich protein 1	CSRP1	4.58E+00	-1.86
P61160	Actin-related protein 2	ACTR2	5.19E+00	-1.93
P50552	Vasodilator-stimulated phosphoprotein	VASP	4.93E+00	-1.97
A0A075B7D0		IGHV1OR15-1	1.37E+00	-1.98
Q14019	Coactosin-like protein	COTL1	3.34E+00	-2.00
O00161	Synaptosomal-associated protein 23	SNAP23	3.44E+00	-2.08
A6NNI4	Tetraspanin	CD9	5.14E+00	-2.23
P62937	Peptidyl-prolyl cis-trans isomerase A	PPIA	5.76E+00	-2.24
Q15942	Zyxin	ZYX	5.35E+00	-2.33
P04040	Catalase	CAT	3.44E+00	-2.37
Q96DA0	Zymogen granule protein 16 homolog B	ZG16B	2.79E+00	-2.37
P61224	Ras-related protein Rap-1b	RAP1B	6.00E+00	-2.45
P16284	Platelet endothelial cell adhesion molecule	PECAM1	4.27E+00	-2.59
O60268-3	Uncharacterized protein KIAA0513	KIAA0513	3.69E+00	-2.71
O14818	Proteasome subunit alpha type-7	PSMA7	3.21E+00	-2.78
P07384	Calpain-1 catalytic subunit	CAPN1	4.35E+00	-2.79
Q15833	Syntaxin-binding protein 2	STXBP2	3.90E+00	-2.92
O15145	Actin-related protein 2/3 complex subunit 3	ARPC3	6.57E+00	-2.92
Q00013	55 kDa erythrocyte membrane protein	MPP1	5.54E+00	-2.99
P10644-2	cAMP-dependent protein kinase type I-alpha regulatory subunit	PRKAR1A	6.44E+00	-3.00
P11169	Solute carrier family 2, facilitated glucose transporter member 3	SLC2A3	4.20E+00	-3.11
Q9NZN3	EH domain-containing protein 3	EHD3	5.35E+00	-3.23
P50148	Guanine nucleotide-binding protein G(q) subunit alpha	GNAQ	6.76E+00	-3.38
Q9UBW5	Bridging integrator 2	BIN2	6.20E+00	-3.90

a EV-prot expression profile was evaluated in SF and PL samples from OJIA patients and PL from control subjects, and comparison of the two datasets was carried out. EV-prots specifically modulated in the comparison of SF_OJIA vs PL_OJIA are shown. EV-prots are listed by decreasing FC value. When protein designation is not available in the Uniprot database, proteins are identified by gene names and/or specific Uniprot IDs.

b Official protein ID.

c Name of protein-coding genes.

d P value adjusted for FDR (False discovery rate). Values expressed as -log10. Adjusted p values lower than 0.01 are considered significant.

e Fold change values expressed as log2. FC values greater than 1 are reported.

Understanding the biological processes in which EV-prots are implicated may help to elucidate disease molecular mechanisms and be instrumental for the identification of new putative biomarkers and therapeutic targets. To help interpret the data biologically and define regulated biological processes in OJIA joints, network and pathway analysis were then carried out on the selected subset of 83 EV-prots found specifically differentially expressed in SF_OJIA vs PL_OJIA samples using STRINGApp and the ShinyGO web server, respectively. Network analysis demonstrated that modulated proteins displayed significantly more interactions than those expected for a random set of proteins of similar size drawn from the genome (PPI enrichment p-value<0.05), indicating their biological connection as a group (**Figures 3D, E**). Pathway analysis showed the significant enrichment (FDR ≤ 0.05) of 547 GO biological processes (283 associated to up- and 263 to down-regulated EV-prots) in SF with respect to PL samples (**Supplementary Table 3**). A selection of the most significantly enriched terms associated to up- and down-regulated EV-prots is depicted in **Figures 3D, E**, respectively. Proteins associated with each selected pathway are listed in **Supplementary Table 4**. The majority of up-regulated EV-prots was implicated in biological processes related to cartilage/bone organization, such as regulation of extracellular matrix (ECM) disassembly and collagen carbolic processes, chondroitin sulfate metabolic processes, connective tissue and skeletal tissue development, regulation of peptidase activity, cell adhesion, motility and migration,. They include collagen alpha-1(II) chain (COL2A1), chondroitin sulfate proteoglycan 4 (CSPG4), fibroblast activation protein (FAP), nidogen-2 (NID2), vitronectin (VTN), low-density lipoprotein receptor-related protein 1 (LRP1), EGF-containing fibulin-like extracellular matrix protein 1 (EFEMP1), follistatin-like protein 1 (FSTL1), protein S100-A8 (S100A8), lymphocyte function-associated antigen-3 (CD58), macrophage colony-stimulating factor 1 (CSF1), and cluster of differentiation 14 (CD14), Conversely, down-regulated EV-prots were mostly involved in the regulation of actin filament polymerization and cytoskeleton organization, including actin-alpha 1 (ACTA1), actin-related protein 2/3 complex subunits (ARPC1B,2,3,4,5), actin-related protein 2 (ACTR2), profilin-1 (PFN1), tropomyosin alpha-3 chain (TPM3), cofilin-1 (CFL1), adenylyl cyclase-associated protein 1 (CAP1), zyxin (ZYX), and vasodilator-stimulated phosphoprotein (VASP). Many down-regulated proteins were implicated in inflammatory and innate immune processes, such as neutrophil, granulocyte, and myeloid leukocyte degranulation, activation, and -mediated immunity, immune effector process, cell secretion, and exocytosis, e.g. platelet endothelial cell adhesion molecule (PECAM1), bridging integrator 2 (BIN2), Src kinase-associated phosphoprotein 2 (SKAP2), and receptor-type tyrosine-protein phosphatase eta (PTPRJ, CD148).

**Figure 4:**
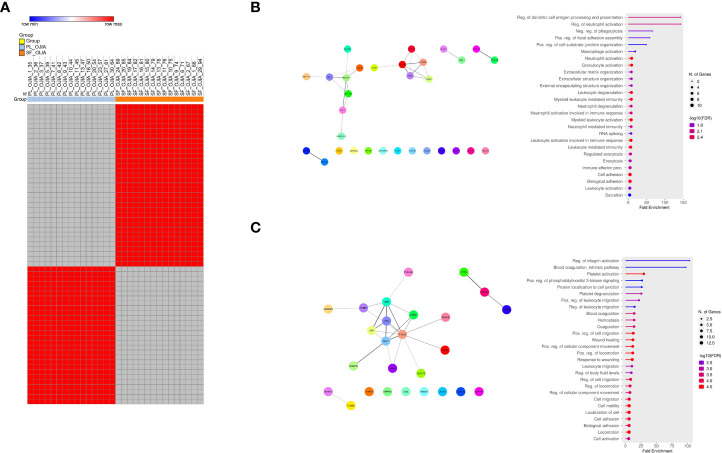
Analysis of the distribution of missing values in paired PL_OJIA vs SF_OJIA samples. Distribution of missing values between the fifteen paired SF and PL samples was assessed by a matched pair binomial test. Exclusively expressed EV-prots were subjected to network and pathway analyses. **(A)** Heat-map representation shows detectable (red color) and missing values (gray color) across samples. Each column corresponds to a sample (indicated on the top side) and each row represents a differentially expressed protein derived from the comparison. The blue and red bars above the columns distinguish the SF and PL groups. Differences with p-value lower than 0.0001 were considered statistically significant. Heat-map was drawn using global coloring. A good association between clusters of EV-prot and the SF and PL groups of samples is shown. Protein designation was made according to the Uniprot database nomenclature, when available. The proteins in position 1 and 45, whose name is missing in the Uniprot database, have Uniprot ID number A0A182DWH4 and P09493-8, respectively. **(B, C)** Network and pathway enrichment analyses were carried out on EV-prots uniquely expressed in SF **(B)** and PL **(C)** samples. Functional interaction networks among EV-prots and GO enriched biological processes are depicted as detailed in the legend of **Figures 3C, D**.

The distribution of missing values between paired SF and PL samples from OJIA patients was then investigated by two-tailed matched pair binomial test to identify EV-prots expressed exclusively in the joints or in the circulation of OJIA patients and thus obtain further insights into the mechanisms of disease development. P value ≤ 0.0001 was considered statistically significant. This analysis identified subsets of 33 and 28 EV-prots exclusively expressed in SF or PL groups, respectively (p-value<0.0001, **Table 3**). Heatmap visualization of EV-prot expression values showed a clear association of significant EV-prots and SF or PL samples (**Figure 4A**), visually confirming the results of the binomial test. Network analysis established a significant connection among some identified EV-prots (PPI p-value<0.05, **Figures 4B, C**). Pathway analysis of these proteins showed the significant enrichment (FDR ≤ 0.05) of 98 GO biological (41 related to EV-prots expressed in SF and 57 in PL) (**Supplementary Table 5**). A selection of the most significantly enriched terms is depicted in **Figures 4B, C**, and the list of EV-prots specifically associated with each pathway is shown in **Supplementary Table 6**. EV-prots expressed exclusively in SF were involved in several innate immune pathways and cell/matrix interactions, including ECM organization, regulation of dendritic cell (DC) antigen processing/presentation and of neutrophil activation, negative regulation of phagocytosis, macrophage, neutrophil, and granulocyte activation, leukocyte degranulation and -mediated immunity, immune effector processes, and cell adhesion, Among them, collagen alpha-1(V) chain (COL5A1), hyaluronan and proteoglycan link protein 1(HAPLN1), integrin alpha-M (ITGAM), integrin subunit alpha x (ITGAX), protein S100-A10 (S100A10), CD74 (HLA class II gamma chain), and low affinity Ig-gamma Fc region receptor II-b (FCGR2B) are critical for the maintenance and the normal metabolism of joint tissues.

**Table 3:** List of EV-prots exclusively expressed in SF_OJIA respect to PL_OJIA samples^a^.

Protein IDs^b^	Protein names	Gene names^c^	Binomial p-values^d^
SF			
A0A182DWH4			6.10352E-05
C4AMC7	Putative WAS protein family homolog 3;WAS protein family homolog 2;WAS protein family homolog 1	WASH3P	6.10352E-05
P31943	Heterogeneous nuclear ribonucleoprotein H;Heterogeneous nuclear ribonucleoprotein H, N-terminally processed	HNRNPH1	6.10352E-05
O95466-2	Formin-like protein 1	FMNL1	6.10352E-05
P04080	Cystatin-B	CSTB	6.10352E-05
P04233-2	HLA class II histocompatibility antigen gamma chain	CD74	6.10352E-05
P08311	Cathepsin G	CTSG	6.10352E-05
P10915	Hyaluronan and proteoglycan link protein 1	HAPLN1	6.10352E-05
P11215-2	Integrin alpha-M	ITGAM	6.10352E-05
P16403	Histone H1.2;Histone H1.4;Histone H1.3	HIST1H1C	6.10352E-05
P19338	Nucleolin	NCL	6.10352E-05
P20702	Integrin alpha-X	ITGAX	6.10352E-05
P20908	Collagen alpha-1(V) chain	COL5A1	6.10352E-05
P31994-5	Low affinity immunoglobulin gamma Fc region receptor II-b	FCGR2B	6.10352E-05
P46940	Ras GTPase-activating-like protein IQGAP1	IQGAP1	6.10352E-05
P48960-2	CD97 antigen;CD97 antigen subunit alpha;CD97 antigen subunit beta	CD97	6.10352E-05
P52943	Cysteine-rich protein 2	CRIP2	6.10352E-05
P60903	Protein S100-A10	S100A10	6.10352E-05
P78324-4	Tyrosine-protein phosphatase non-receptor type substrate 1	SIRPA	6.10352E-05
Q09666	Neuroblast differentiation-associated protein AHNAK	AHNAK	6.10352E-05
Q15029-2	116 kDa U5 small nuclear ribonucleoprotein component	EFTUD2	6.10352E-05
Q15113	Procollagen C-endopeptidase enhancer 1	PCOLCE	6.10352E-05
Q15424	Scaffold attachment factor B1	SAFB	6.10352E-05
Q1KMD3	Heterogeneous nuclear ribonucleoprotein U-like protein 2	HNRNPUL2	6.10352E-05
Q7Z6I6	Rho GTPase-activating protein 30	ARHGAP30	6.10352E-05
Q8N8A2	Serine/threonine-protein phosphatase 6 regulatory ankyrin repeat subunit B	ANKRD44	6.10352E-05
Q96C19	EF-hand domain-containing protein D2	EFHD2	6.10352E-05
Q96CX2	BTB/POZ domain-containing protein KCTD12	KCTD12	6.10352E-05
Q9BTT0	Acidic leucine-rich nuclear phosphoprotein 32 family member E	ANP32E	6.10352E-05
Q9H1E3	Nuclear ubiquitous casein and cyclin-dependent kinase substrate 1	NUCKS1	6.10352E-05
Q9UKM9-2	RNA-binding protein Raly	RALY	6.10352E-05
Q9Y3X0	Coiled-coil domain-containing protein 9	CCDC9	6.10352E-05
Q9Y639-1	Neuroplastin	NPTN	6.10352E-05
PL			
P01137	Transforming growth factor beta-1;Latency-associated peptide	TGFB1	6.10352E-05
B1AMS2	Septin-6	SEPT6	6.10352E-05
O75954	Tetraspanin-9;Tetraspanin	TSPAN9	6.10352E-05
C9JAI6	CKLF-like MARVEL transmembrane domain-containing protein 5	CMTM5	6.10352E-05
F6VVT6	P-selectin	SELP	6.10352E-05
J3KQ66	Reelin	RELN	6.10352E-05
O00194	Ras-related protein Rab-27B	RAB27B	6.10352E-05
O43157	Plexin-B1	PLXNB1	6.10352E-05
P00918	Carbonic anhydrase 2	CA2	6.10352E-05
P02775	Platelet basic protein; Connective tissue-activating peptide III; TC-2; Connective tissue-activating peptide III(1-81);Beta-thromboglobulin	PPBP	6.10352E-05
P08514	Integrin alpha-IIb; Integrin alpha-IIb heavy chain; Integrin alpha-IIb light chain, form 1;Integrin alpha-IIb light chain, form 2	ITGA2B	6.10352E-05
P09493-8			6.10352E-05
P13716	Delta-aminolevulinic acid dehydratase	ALAD	6.10352E-05
P14770	Platelet glycoprotein IX	GP9	6.10352E-05
P18054	Arachidonate 12-lipoxygenase, 12S-type	ALOX12	6.10352E-05
P40197	Platelet glycoprotein V	GP5	6.10352E-05
P67936-2	Tropomyosin alpha-4 chain	TPM4	6.10352E-05
Q14247-3	Src substrate cortactin	CTTN	6.10352E-05
Q5T1B5	Type I inositol 1,4,5-trisphosphate 5-phosphatase	INPP5A	6.10352E-05
Q14644	Ras GTPase-activating protein 3	RASA3	6.10352E-05
Q15555-4	Microtubule-associated protein RP/EB family member 2	MAPRE2	6.10352E-05
Q15746-5	Myosin light chain kinase, smooth muscle; Myosin light chain kinase, smooth muscle, deglutamylated form	MYLK	6.10352E-05
Q3ZCW2	Galectin-related protein; Galectin	LGALSL	6.10352E-05
Q99436	Proteasome subunit beta type-7	PSMB7	6.10352E-05
Q9BX67	Junctional adhesion molecule C	JAM3	6.10352E-05
Q9H4B7	Tubulin beta-1 chain	TUBB1	6.10352E-05
Q9HBI1-3	Beta-parvin	PARVB	6.10352E-05
Q9HCN6-2	Platelet glycoprotein VI	GP6	6.10352E-05

a The distribution of missing values between paired SF and PL samples from OJIA patients was evaluated by two-tailed matched pair binomial test. EV-prots exclusively expressed in each sample are shown. When protein designation is not available in the Uniprot database, proteins are identified by gene names and/or specific Uniprot IDs.

b Official protein ID.

c Name of protein-coding genes.

d Differences with p-value lower than 0.0001 were considered statistically significant.

These results characterize the biological processes regulated by EV-prots within the joints of OJIA patients at an early stage of the disease and identify EV-prots potentially implicated in disease pathogenesis, representing new early putative joint-specific biomarkers of OJIA development.

### Analysis of EV-prot expression profiles in PL samples differentiates new-onset OJIA patients from control children

**Figure 5:**
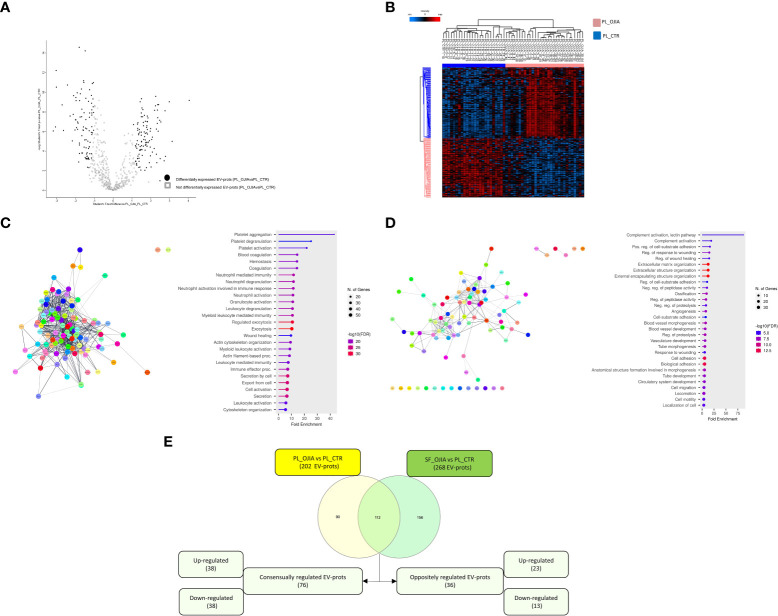
Comparative analysis of EV-prot expression profiles between PL_OJIA and PL_CTR specimens. EV-prot expression profiles were compared between the full sets of PL samples from new-onset OJIA patients and CTR children, and differences in the expression patterns were defined. Differentially expressed EV-prots were subjected to network and functional enrichment analyses. **(A)** Volcano plot shows differentially expressed EV-prots between the two groups of samples. EV-prot expression levels are indicated as described in the legend of **Figure 3B**. On the left EV-prots downregulated and on the right those up-regulated in PL_OJIA respect to PL_CTR samples are shown. **(B)** Heat-map representation shows unsupervised hierarchical clustering analysis of significantly differentially expressed EV-prots in PL_OJIA vs PL_CTR samples. EV-prot expression levels are indicated as described in the legend of **Figure 3B**. The pink and blue bars above the columns distinguish the OJIA and CTR groups. The optimal association between two main clusters of EV-prots (indicated by the pink and blue bar on the left side) identified by the unsupervised hierarchical method and the PL_OJIA and PL_CTR groups of samples is shown. **(C, D)** Network and pathway enrichment analyses were carried out on up- **(C)** and down- **(D)** regulated EV-prots. Interaction networks among EV-prots and GO enriched biological processes are depicted as described in the legend of **Figures 3C, D**. **(E)** Venn diagram shows the number of common and exclusive differentially expressed EV-prots detectable in paired SF_OJIA and PL_OJIA respect to PL_CTR specimens. Each comparison is depicted by a distinct color. Common EV-prots displaying consensual and opposite modulation are indicated. Both up- and downregulated EV-prots were considered. The significance of the overlapping was estimated with hypergeometric statistics.

Proteome evaluation was then carried out on the full cohort of PL_OJIA specimens compared to PL_CTR samples to identify EV-prots that could represent early putative diagnostic biomarkers. As shown by the Volcano plot depicted in **Figure 5A**, patients and control children expressed clearly distinct EV-prot patterns, which was confirmed by unsupervised hierarchical clustering analysis shown in the heat map (**Figure 5B**). We detected significantly altered expression levels of a total of 202 EV-prots between groups, of which 110 were upregulated and 92 downregulated in patient specimens, suggesting specific disease-related changes in the expression levels of circulating EV-prots. The complete list of differentially regulated EV-prots is reported in **Supplementary Table 7**.

Network analysis demonstrated that modulated proteins displayed significant functional interactions (PPI enrichment p-value<0.05) (**Figures 5C, D**). Pathway analysis identified 1099 significantly enriched (FDR ≤ 0.05) biological processes (802 associated to up- and 297 to down-regulated EV-prots) (**Supplementary Table 8**). A selection of the processes with the most significant enrichment score and associated proteins are reported in **Figures 5C, D** and **Supplementary Table 4**. Among them, several are related to inflammation and cytoskeleton organization, including: (i) neutrophil, granulocyte, and myeloid leukocyte degranulation, activation, and -mediated immunity, immune effector processes, cell secretion, exocytosis and actin cytoskeleton organization for up-regulated proteins; (ii) complement activation, ECM organization, regulation of peptidase activity and proteolysis, angiogenesis, cell adhesion, migration, and motility for downregulated proteins. Proteins mostly involved in these processes include several Ras-related proteins (e.g. Rab-11B, RAB8A, RAP1B), Rab GDP dissociation inhibitor beta (GDI2), heat shock protein A8 (HSPA8), calpain-1,2 catalytic subunits (CAPN1), catalase (CAT), glutathione S-transferase omega-1 (GSTO1), and annexin A1 (ANXA1), matrix-remodeling-associated protein 5 (MXRA5), carboxypeptidase B2 (CPB2), respectively.

Interestingly, some of the EV-prots differentially modulated in PL_OJIA vs PL_CTR overlapped with those observed in SF_OJIA vs PL_CTR. Common and exclusive differentially expressed proteins were visualized by the Venn diagram depicted in **Figure 5E** following comparison of the two datasets. Among common EV-prots, 76 displayed consensual regulation; specifically 38 up-regulation, e.g. HSPB1, HSPA1B, platelet glycoprotein 4 (CD36), proto-oncogene tyrosine-protein kinase Src (SRC), lyn proto-oncogene (LYN), peroxiredoxin-1 (PRDX1), thioredoxin (TXN), glutathione S-transferase P (GSTP1), pyruvate kinase m1/2 (PKM), glyceraldehyde-3 phosphate dehydrogenase (GAPDH), L-lactate dehydrogenase a chain (LDHA), triosephosphate isomerase (TPI1), phosphoglycerate kinase 1 (PGK1), and 38 down-regulation, e.g. complement factor H-related protein 1 (CFHR1), mannose-binding protein C (MBL2), mannan binding lectin serine peptidase 1 (MASP1), collectin-10 (COLEC10), insulin-like growth factor-binding protein 3 (IGFBP3) (**Supplementary Tables 2, 7** underlined proteins), potentially representing disease state indicators measurable at both the systemic and local levels able to discriminate new-onset OJIA patients from healthy children.

**Figure 6:**
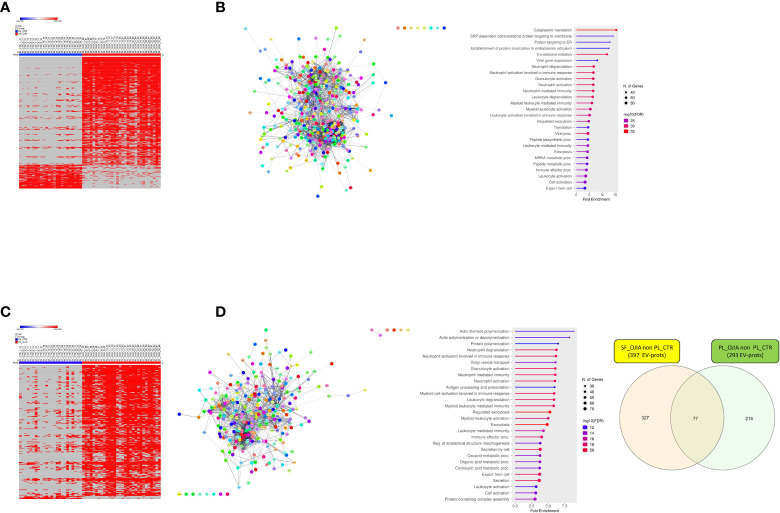
Analysis of the distribution of missing values in SF_OJIA vs PL_CTR and PL_OJIA vs. PL_CTR. Distribution of missing values between the full sets of OJIA and CTR samples was assessed by a two sample Z-test for proportions, and EV-prots exclusively expressed in SF_OJIA and PL_OJIA were subjected to network and functional enrichment analyses. **(A, C)** Heat-map representations show detectable and missing values across samples. Each column corresponds to a sample (indicated on the top side) and each row represents a differentially expressed protein derived from the comparison. The blue and red bars above the columns distinguish the OJIA and CTR groups. Proteins with z-test scores lower than -4.0 or higher than 4.0 (p<0.00005) were considered to have statistically significant differences between groups. Heatmap was drawn using global coloring. A good association between clusters of EV-prots exclusively expressed in SF_OJIA **(A)** and PL_OJIA **(C)** respect to PL_CTR and vice versa is shown. **(B, D)** Network and pathway enrichment analyses were carried out on EV-prots uniquely expressed in SF **(B)** and PL **(D)** samples respect to PL_CTR. Functional interaction networks among EV-prots and GO enriched biological processes are depicted as detailed in the legend of **Figures 3C, D**. **(E)** Venn diagrams show the number of common and exclusive EV-prots detectable between the comparisons of paired SF_OJIA and PL_OJIA respect to PL_CTR specimens. Each comparison is depicted by a distinct color. The significance of the overlapping was estimated with hypergeometric statistics.

Two sample Z-test for proportions was used to detect EV-prots whose distribution of missing values resulted statistically significantly different between the full cohort of OJIA and CTR sample groups. EV-prots with z-scores lower than -4.0 or higher than 4.0 (p<0.00005) were considered significantly exclusively expressed. This analysis showed that a total of 410 and 294 EV-prots were exclusively expressed in SF_OJIA or PL_OJIA, respectively, whereas 90 and 7 were exclusively expressed in PL_CTR as compared to SF_OJIA or PL_OJIA (**Supplementary Table 9**). Heat map analysis showed EV-prot association with OJIA patients or CTR subjects, visually confirming the results of the z-test (**Figures 6A, C**). Network analysis carried out on the EV-prots expressed exclusively in SF_OJIA (**Figure 6B**) and PL_OJIA (**Figure 6D**) showed significant interactions among these proteins (PPI p-value<0.05). Pathway analysis revealed the significant enrichment (FDR ≤ 0.05) of a total of 821 biological processes related to EV-prots exclusively expressed in SF and 613 to those exclusively expressed in PL, whereas 288 were enriched with proteins expressed in PL_CTR and not in SF (**Supplementary Tables 10, 11**). In contrast, no biological pathway associated with the 7 proteins expressed in PL_CTR and not in PL_OJIA was found. A selection of the most significantly enriched GO terms and the list of associated proteins are shown in **Figures 6B, D** and **Supplementary Table 6**. Proteins identified in both SF and PL were involved in inflammatory responses, being mostly related to neutrophil, granulocyte, and leukocyte degranulation, activation, and mediated immunity, regulated exocytosis, and immune effector processes. Proteins detectable in SF were also involved in cytoplasmic translation, protein targeting to membrane and endoplasmic reticulum, mRNA and peptide metabolic processes, whereas a significant number of those detectable in PL are critical for cytoskeleton organization being mainly implicated in actin polymerization, Golgi vesicle transport, antigen processing and presentation. CRP, serum amyloid A (SAA1), cartilage intermediate layer protein 1 (CILP), RALB, RAB10, Rho GTPase-activating protein 18 (ARHGAP18), ras-related proteins (RAB1A, RAB5B, RAB14), heat shock 70 kDa protein 4 (HSPA4), plasminogen-activator inhibitor 1 (SERPINE1, B6,9), SLAM family member 5 (CD84), signal transducer and activator of transcription 3 (STAT3), spleen associated tyrosine kinase (SYK), triggering receptor expressed on myeloid cells-like transcript-1 (TREML1), and drebrin 1 (DBN1) were some of the EV-prots identified in PL_OJIA vs PL_CTR samples. As shown by the Venn diagram depicted in **Figure 6E**, 77 EV-prots were shared by the PL_OJIA and SF_OJIA datasets, among which Ras homolog gene family member a (RhoA), cell division control protein 42 homolog (CDC42), Ras-related protein-A (RALA), RAB5C, RAB35, ITGA5, basigin (BSG, CD147), and leukocyte elastase inhibitor (SERPINB1) (**Supplementary Table 9**, underlined proteins), suggestive of a generalized disease state indicator.

Taken together, these data demonstrate that the expression levels of several EV-prots or exclusively expressed EV-prots subsets can differentiate new-onset OJIA patients from CTR children with potential diagnostic value.

### Co-expression network analysis applied to EV-prot signature and patient clinical parameters

**Figure 7:**
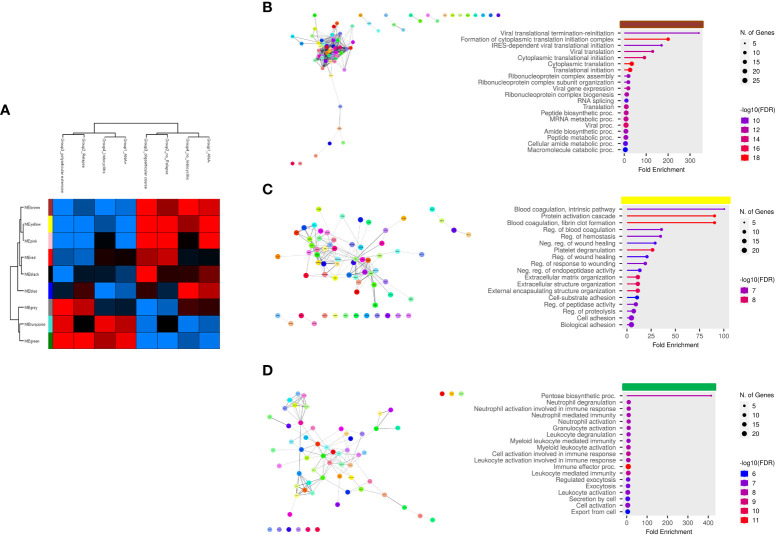
Weighted correlation co-expression network analysis of EV-prot datasets in SF_OJIA samples. WGCNA was carried out on the 30 SF_OJIA samples with a specific software R package in the Perseus platform to construct co-expression modules functionally associated with four selected patient clinical and laboratory parameters. Proteins were grouped based on the correlation of their expression with the clinical parameters used. **(A)** Heat map representation shows the weighted relationship between nine protein modules and the four patient clinical parameters. Each row represents a module and each column a clinical group. Each module is represented by a specific color. Modules are distinguished by different colors. The color scale shows module-clinical groups Pearson correlation, with red for positive correlation, blue for negative correlation, and black for equal correlation. The hierarchical cluster dendrogram is displayed at the top of the plot. **(B–D)** Functional interaction networks and GO biological process enrichment analyses were carried out on selected EV-prot modules. as detailed in the legend of **Figures 3C, D**. Selected modules are indicated by the horizontal colored bars on top of the graphs showing the most relevant enriched GO terms.

**Figure 8:**
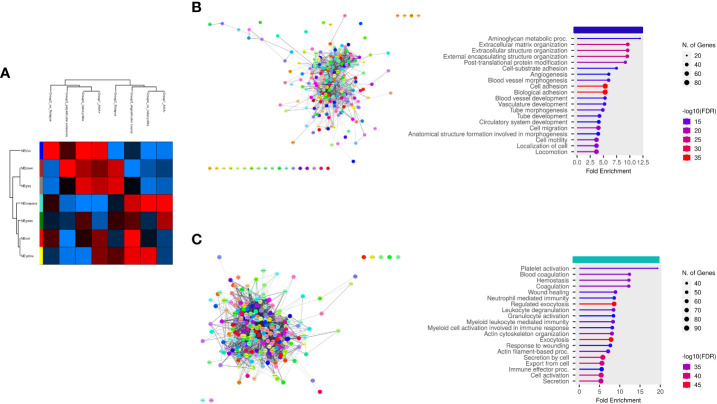
Weighted correlation network analysis of EV-prot datasets in PL_OJIA samples. WGCNA was carried out on the 30 PL_OJIA samples to construct Coexpression Modules functionally associated with selected patient clinical and laboratory parameters. **(A)** Heat-map representation shows the interactive relationships of seven identified co-expression modules with the four OJIA clinical parameters. Results are indicated as described in the legend of **Figure 7A**. **(B, C)** Functional interaction networks and GO biological process enrichment analyses were carried out on selected EV-prot modules. as detailed in the legend of **Figures 3C,D**. Selected modules are indicated by the horizontal colored bars on top of the graphs showing the most relevant enriched GO terms. .

Finally, we aimed at establishing the existence of an association between EV-Prot expression profile of SF-OJIA and PL_OJIA samples collected at disease onset and patient clinical parameters, such as ANA positivity, disease relapse, polyarticular extension, and iridocyclitis development within the 2 years of follow-up. WGCNA was run on the full cohorts of SF- and PL-derived EV-prot datasets characterized in newly-diagnosed OJIA patients to identify biologically significant clusters of proteins (modules) associated to the selected clinical parameters, as described in Ref (60). We reasoned that these EV-prot clusters could potentially represent early indicators of patient outcome. A heatmap was drawn based on the interactive relationships of identified co-expression modules with the OJIA traits (**Figures 7**, **8**). The different clinical groups were clustered by hierarchical clustering and visualized by a dendrogram on the bases of their relation with different EV-prot modules. For SF_OJIA, WGCNA revealed a total of 9 EV-prot coexpression modules (each distinguished by a specific color) associated with the different clinical parameter considered, whereas for PL_OJIA, we identified a total of 7 EV-prot modules. With regard to SF samples (**Figure 7A**), the brown, yellow and green modules seemed the most interesting, being the ones that best discriminated the two clusters into which the clinical groups are divided. Modules brown and yellow were found significantly correlated to negativity for ANA, oligoarticular course, no iridocyclitis, and no relapse, whereas the green one was highly associated with positivity for ANA, polyarticular extension, iridocyclitis development, and relapse. With regard to PL samples (**Figure 8A**), the most interesting modules seemed to be the blue, which correlates with positivity for ANA, iridocyclitis development, and no relapse, and the turquoise, highly associated to ANA-, oligoarticular course, and no iridocyclitis. The list of proteins belonging to selected modules is shown in **Supplementary Table 12**.

PPI network analysis and GO annotation of the most significant co-expression modules were then carried out to assess interconnectivity and explore their functions (**Figures 7B–D**, **8B, C**). A large number of proteins within each module was connected as a group. Proteins associated to the most relevant functional pathways are shown in **Supplementary Table 13**. In SF (**Figures 7B–D**), the brown module contained EV-prot related to ribonucleoprotein complex assembly, organization and biogenesis, mRNA metabolic processes, and protein translation. The most represented proteins in this pathway included several heterogeneous nuclear ribonucleoproteins (HNRNPs) (e.g. HNRNPA2B1, HNRNPD, HNRNPM, HNRNPH1, and HNRNPA3), high mobility group protein B1 (HMGB1), nucleolin (NCL), and proliferating cell nuclear antigen (PCNA). The yellow module contained proteins involved in the regulation of blood coagulation, ECM organization, regulation of protease activation and proteolysis, such as a few collagen molecules (COL2A1, COL6A1,2,3, COL11A1), tetranectin (CLEC3B), attractin (ATRN), lumican (LUM), osteomodulin (OMD), fibulin-1 (FBLN1), dentin matrix acidic phosphoprotein 1 (DMP1), fibronectin (1FN1), cartilage acidic protein 1 (CRTAC1), transforming growth factor-beta-induced protein (TGFBI), CD109 antigen (CD109), and neuropilin-1 (NRP1). The green module contained proteins with known regulatory effects on inflammatory/immune processes, such as granulocyte, neutrophil, and myeloid cell degranulation, activation and mediated immunity and immune effector processes, including SERPINB1, HSPA1B, immune costimulatory protein b7-h3 (CD276), neutrophil gelatinase-associated lipocalin (LCN2), low affinity Ig-gamma Fc region receptor III-A (FCGR3A), complement components C5 and C6, protein S100-A12, CRP, FAP, matrimetalloproteinase-9 (MMP9), myeloperoxidase (MPO), and secreted phosphoprotein 1 (SPP1).

In the PL (**Figures 8B, C**), biological processes of the blue module were mainly enriched in proteins related to glycosaminoglycan metabolic processes, ECM organization, post translational protein modification, angiogenesis, cell adhesion, migration and motility, whereas the turquoise module revealed proteins involved in the regulation of granulocyte, neutrophil, and myeloid cell degranulation, activation and mediated immunity, exocytosis, actin cytoskeleton organization, response to wounding, Proteins in the blue module include ATRN, FBLN1, CLEC3B, NRP1, TGFB1, CD109, CSF1, macrophage colony-stimulating factor 1 receptor (CSF1R), thrombospondin-2 (THBS2), fibrillin 1 (FBN1), endosialin (CD248), CD166 antigen (ALCAM), CD44 antigen, lymphocyte cytosolic protein 1(LCP1), lipopolysaccharide-binding protein (LBP), and, fibrinogen-like protein 1 (FGL1). Proteins in the turquoise module included SERPINB1, STAT3, SYK, LYN, HSPAIB, HSPB1, CD36, BSG, junctional adhesion molecule C (JAM3), ITGA5, PRDX1, SRC, PKM, aldolase A (ALDOA), solute carrier family 2 (SLC2A3), glucose-6-phosphate isomerase (GPI), disintegrin and metalloproteinase domain-containing protein 10 (ADAM10), P-selectin (SELP), and CD226 antigen (DNAM-1).

We can conclude that a few clusters of EV-prots highly associated with patient clinical parameters can discriminate subgroups of patients with different disease courses at 2 years of follow up, suggesting their potential as early biomarkers of disease outcome.

## Discussion

This is the first study that comprehensively profiles EV proteome in OJIA patients at disease onset and assesses its longitudinal association with clinical parameters at the 2 year follow-up to find biomarkers that could be indicative of outcome. Our results define specific EV-prot signatures in SF and PL samples potentially representing new early molecular indicators of OJIA development able to discriminate new-onset OJIA patients from control children. Moreover, they identify EV-prot clusters that associate with specific clinical features stratifying subgroups of patients with different disease course, yielding a framework for the discovery of candidate biomarkers for the disease and novel targets for tailored therapy.

High-throughput proteomic techniques such as LC-MS has increasingly been utilized to profile biological samples, facilitating the search for new biomarkers of clinical use in rheumatic diseases (61). Over the last years, many studies have focused on the proteome analysis of EVs released in biofluids as a minimally invasive approach to elucidate disease pathogenic mechanisms (30, 31) and identify novel diagnostic and prognostic markers (33, 35, 36, 62). EV-associated proteins present several advantages respect to “free” proteins for new biomarker identification (32). Interrogation of the proteome in unfractionated biologic fluids is hindered by their large protein concentration dynamic range, which prevents detection of proteins present at low concentration (which are likely the proteins of interest) (32). In contrast, EV-prots are enriched and protected from degradation, characteristics that improve detectability while reducing the probability of false negative results. In addition, because EV cargo loading is an active and strictly regulated mechanism, EV-prots highly reflect the type and state of the cell of origin that vary as a function of the pathological situation (29, 30), providing more specific markers.

Although previous reports have analyzed the profile of “free” proteins in various biofluids (e.g. PL, SF, aqueous humor, tears) of patients with different JIA subtypes (16, 37–40, 63), to our knowledge no study has investigated EV-prots and their potential as biomarkers in these diseases. Given EV role in immune and inflammatory responses (20, 22–24, 64), it is conceivable that analysis of their protein cargo in the joints early in the disease process may help to elucidate immune-mediated molecular mechanisms underlying OJIA pathophysiology and yield early candidate biomarkers of disease development/activity and predictors of outcome useful for assisting clinical decision-making. The finding that an elevated number of EV-prots were differentially or exclusively expressed in SF *vs* matched PL samples from newly-diagnosed OJIA patients probably reflect the enrichment of distinct EV populations endowed with specific biologic functions or of distinct nature (29, 65) related to the diverse cellular composition in the joints respect to the circulation. These results are in line with our previous findings demonstrating different microRNAs patterns in EVs released in, and mononuclear cells from, SF and PB samples of new-onset OJIA patients (45, 66), suggesting specific fingerprints of joint disease.

Several identified proteins were enriched in biologic processes essential for joint homeostasis but whose disruption might be implicated in arthritis development. The observed increase in the abundance of various collagen and non-collagenous ECM components (glycoproteins, proteoglycan, and integrins) may be relevant for OJIA joint pathology contributing to synovial tissue remodeling, cell-cell/cell-ECM interaction, and myeloid cell/FLS migration and destructive activities (67, 68). Most of these proteins have been previously found overexpressed in ST, SF, and cartilage of RA (69–71), OA (72–77), and/or JIA patients (78), correlating with disease severity (69, 79–82), and regarded as promising diagnostic/prognostic biomarkers (72–74) or new targets of treatment (76, 83–85). Downregulation in SF*vs*PL of EV-prots with a role in the regulation of actin cytoskeleton polymerization and remodeling (86) may also provide potential markers of cartilage degeneration and disease development. Decreased expression of some of them (e.g. PFN1, CFL1, and VASP) was suggested to be involved in RA pathogenesis and have diagnostic or therapeutic potential (87, 88).

A large subset of EV-prots overexpressed in SF samples was linked to processes involved in myeloid cell degranulation, activation, and mediated immunity, suggesting a strong inflammatory and innate immune signature which supports our earlier findings of a pivotal role of innate immunity in OJIA pathogenesis (6, 7, 89, 90). S100A8 and S100A10, two members of the S100A protein family mainly secreted by activated myeloid cells, are particular relevant given to their role as endogenous DAMPs, triggering innate immunity activation and inflammatory reactions (91). High S100A8 expression has been detected in ST or cartilage and in various biofluids of patients with adult arthritis (91, 92) and systemic JIA (40, 40, 63, 93) and proposed as candidate diagnostic biomarkers (40, 91, 94, 95) or predictors of response to treatment/flares (93). Our findings extend these information demonstrating S100A8 overexpression also in SF-derived EVs from OJIA patients at onset and suggesting its potential as a marker of disease development. Of relevance is also the overexpression of CD58, CSF1, CD74, and FCGR2B, which have been implicated in RA and OA pathogenesis (96–99) and regarded as potential therapeutic targets (96, 100, 101). The demonstration of CD14 overexpression in EVs from SF_OJIA is in line with data by Foers et al. in RA (31), suggesting that monocytic cells represent an important source of EVs in SF from both adult and juvenile arthritic patients. On the other hand, downregulation in SF of a subset of EV-prots endowed with inhibitory activities on macrophage and neutrophil proinflammatory functions (102, 103) suggest inhibition of processes involved in the resolution of inflammation. Among them, PECAM1 and PTPRJ were shown to play a role in the protection against arthritis (104) and exert anti-fibrotic effects (105), respectively. Collectively, these findings provide novel information on EV role in synovitis and joint damage in OJIA and define immunogenic and pro-/anti-inflammatory EV-prots likely to mediate these effects which may represent new candidate early biomarkers of disease development and targets of therapy. The evidence of similar alterations in protein patterns in OJIA and adult arthritides is intriguing because OJIA is a form of arthritis seen only in children (8), raising the possibility of common early molecular pathogenetic mechanisms in these conditions.

An important result of this study is the identification in PL samples of a panel of EV-prots able to differentiate OJIA patients at onset from age/gender-matched CTR children, probably representing early diagnostic biomarkers. Discovery of EV-based biomarkers in PL is preferable respect to SF given the easier accessibility of this fluid. EV-prots overexpressed in PL_OJIA were significantly enriched in biological processes related to inflammation, antigen processing/presentation, cell adhesion/migration, and cytoskeleton organization. Our data extend previous findings showing the abundance of two acute phase proteins, CRP and SAA1, as “free” molecules in the circulation of JIA patients. Increased CRP serum levels in the first 6 months of disease have been associated with OJIA polyarticular course, making this protein a candidate predictor of extension (9). Higher SAA1 serum levels were demonstrated in JIA patients vs healthy CTR correlating with the number of active joints, iridocyclitis development, and high CRP and ESR expression, suggesting its potential as a disease activity marker (63, 106). Our results point to a critical role of these molecules also as biomarkers for early OJIA diagnosis. A large group of EV-prots that we found overexpressed in PL_OJIA comprises proteins not previously reported in JIA but proposed as potential diagnostic biomarkers, predictors of outcome, and/or therapeutic targets in RA and OA. Of interest are several members of the Rho (107, 108) and Rab (109, 110) small GTPase, the HSP (111), and the serpin protease inhibitor (112, 113) families. Attractive as putative OJIA diagnostic biomarkers are also CILP, BSG, CD84, and STAT3, given to their roles in immunity, cell-matrix interaction, and inflammatory joint destruction (114–120). Importantly, we identified a group of overexpressed EV-prots not previously described in other arthritic conditions, raising the possibility that they may be specific for OJIA. TREML1, CAPN1, and DBN1 seem the most relevant based on their functions in immune responses, cytoskeletal remodeling, cell adhesion/migration and association with chronic inflammatory and autoimmune diseases (121–124).

Inadequately controlled complement activation during inflammation may be involved in the pathogenesis of various autoimmune and inflammatory diseases (125), including arthritis (126). Interestingly, several EV-prots downregulated in PL from OJIA *vs* CTR children were enriched in processes related to inhibition of complement activation. Among them, CFHR1, MBL2, MASP1, and CPB2 were reported to play protective roles against joint inflammatory injury (127–134). Other proteins that we found downregulated are endowed with anti-inflammatory, anti-fibrotic, and cartilage-protecting properties, e.g. ANXA1 (31, 135–137), MXRA5 (138) and IGFBP3 (139). We hypothesize that downregulation of specific EV-prots in the circulation of OJIA patients may be a mechanism underlying OJIA development and progression and have potential diagnostic relevance.

Particularly intriguing is the observation that many EV-prots are consensually modulated in PL and SF of OJIA patients respect to CTR, suggestive of disease-specific signatures measurable at both systemic and local levels and potentially amenable to clinical utilization in disease diagnosis. Reactive oxygen and nitrogen species accumulate in the hypoxic inflamed joints (89, 140) leading to oxidative stress which contributes to synovitis and joint damage (141, 142). The observation of increased levels of various enzymes with a role in cellular redox homeostasis both in the joint and circulation is noteworthy, suggesting a positive feedback regulatory mechanism in response to oxidative stress. Another general response to conditions of reduced oxygenation is represented by dysregulation of proteins involved in energy metabolisms. Interestingly, several glycolytic enzymes were overexpressed not only in SF_OJIA but also in PL_OJIA respect to CTR suggesting both local and systemic glycolytic reprogramming. These data are consistent with and extend previous evidence showing the crosstalk between metabolic changes and inflammatory/immune responses in RA (143, 144). Several identified proteins are currently being studied in animal models of autoimmune diseases as attractive targets for therapy (143, 145–147).

Finally, we demonstrated by WGCNA that EV-proteome findings could be of potential utility for OJIA patient stratification. WGCNA is a system biology method for identifying modules of biologically relevant genes or proteins highly correlated with clinical traits (60), which has been used in rheumatology to find gene correlations with RA and OA status (57, 148, 149). We defined EV-prot modules in SF and PL that highly associated with specific clinical parameters at the 2 year follow-up, clustering subgroups of patients with different clinical outcomes at disease presentation. With regard to SF, modules correlated to ANA negativity, oligoarticular course, no iridocyclitis, and no relapse were enriched in proteins related to ribonucleoprotein complex assembly, organization, and biogenesis, such as HNRNPs (150), which function as autoantigens (151–153) and have potential as targets of therapy in RA (154), and nuclear proteins acting in chromatin remodeling (e.g. HMGB1 and NCL). High levels of HMGB1 have been detected in ST/SF of RA and JIA patients and shown to correlate with early disease onset/activity (90, 155) and contribute to bone remodeling (156), representing an inflammatory marker and potential therapeutic target (155, 157). Other relevant EV-prots belonging to these modules were involved in the regulation of ECM organization and cell adhesion/migration (e.g. FN1 and NRP1) and in synovial inflammation, fibrosis, hyperplasia, angiogenesis, and/or joint degradation (e.g. CD109, CRTAC1, and LUM) and proposed as putative development/progression biomarkers or potential therapeutic targets in RA and/or OA (77, 158–162). No data on these proteins have been previously reported in JIA. On the other hand, proteins associated to ANA positivity, polyarticular extension, iridocyclitis development, and relapse were endowed with stimulatory effects on inflammatory/innate immune responses. Among them, some have been previously linked to JIA pathogenesis and severity and regarded as potential prognostic biomarkers, such as CRP (9), MMP9 (163), SPP1 (7, 164, 165), and MPO (166). Interestingly, MPO was reported to be highly enriched in neutrophil-derived EVs from the SF of RA patients (31) and suggested to mediate their destructive effects in RA. The association of these EV-prots with more aggressive OJIA features corroborate previous observations indicating that a high level of inflammation is the major determinant of arthritis progression (9).With regard to PL, the proteins mostly correlated with positivity for ANA, no relapse, and iridocyclitis development were related to ECM organization, cell adhesion/migration, and angiogenesis, including glycoproteins/receptors expressed in FLSs and leukocytes infiltrating the RA/OA synovium (e.g. FBN1, CD248, CD44) and known regulators of inflammatory responses and osteoclastogenesis, such as CSF1/CSF1R, LBP, and FGL1. These proteins have been regarded as candidate biomarkers of disease activity and prognosis or targets of treatment in adult arthritis (96, 167–171). Finally, proteins correlated with negativity for ANA, oligoarticular course, and no iridocyclitis were involved in innate immune cell functions, actin cytoskeleton organization, and response to wounding, e.g., various regulators of glucose transport and metabolism previously associated with RA pathogenesis and regarded as putative therapeutic targets (144), SELP, involved in inflammatory cell recruitment into the RA joint (172–174), and CD226, associated with susceptibility to JIA and proposed as a candidate risk factor for the disease (175).

In conclusion, this study provides mechanistic insights into disease pathophysiology improving our understanding of EV contribution to it. Our results highlight the suitability of EV-prot analysis as a method to derive candidate biomarkers for earlier disease detection and patient stratification and novel molecular targets for further investigation in OJIA, which could possibly help tailoring therapeutic interventions at disease onset paving the way to personalized therapy. We acknowledge that this study has some limitations, such as the lack of comparison of EV-prot expression levels between SF samples from OJIA patients and control subjects. Unfortunately, this issue cannot be addressed due to ethical reasons. The potential confounding effect of different medications, that was not addressed in the present report because of the relatively small sample size of the patient cohort, will be assessed in a new prospective follow-up study carried out on an independent larger cohort of patients which will allow to determine the generalizability of our novel biomarkers on new cases and their ability to predict the risk of disease relapse, extension, and irydocyclitis occurrence in OJIA utilizing machine learning methods (176–178).

## Data availability statement

The datasets presented in this study can be found in online repositories. The names of the repository/repositories and accession number(s) can be found below:PXD039166 (PRIDE) (179).

## Ethics statement

The studies involving human participants were reviewed and approved by the Ethics Committee of the Region Liguria (Approval 165/2018), the Ethics Committee Milano Area 2 (Approval 639/2019), and the Ethics Committee ASL Lecce (Approval N° 36/2019), and the procedures were carried out according to the approved guidelines and in adherence to the general ethical principles set forth in the Declaration of Helsinki. Written informed consent to participate in the study was obtained from the parents or the patient’s legal guardian prior to sample collection.

## Author contributions

FR participated in the experiment set up, acquired the results, contributed to bioinformatics analysis and interpretation of the results, and provided research funding; MB and AP performed the proteomic profiling experiments, acquired the results, and contributed to bioinformatics analysis and interpretation of the results; CR and SP processed patient samples, carried out EV isolation and characterization, EV-prot extraction and contributed to data analysis; DC contributed to bioinformatics data analysis and interpretation of the results; CT provided patient clinical information; GF, ACi, AR, and ACo recruited OJIA patients, collected synovial fluid and blood samples, and provided clinical discussion of data; AE revised the manuscript; MCB conceptualized the study, supervised the research, contributed to data analysis and interpretation, provided research funding, and wrote the manuscript. All authors contributed to the article and approved the submitted version.
