# Supplementary figures and images for: Proteomic profiling of extracellular vesicles in synovial fluid and plasma from Oligoarticular Juvenile Idiopathic Arthritis patients reveals novel immunopathogenic biomarkers
Source: Front Immunol. 2023 Apr 27;14:1134747. doi: 10.3389/fimmu.2023.1134747 (PMC10186353; doi:10.3389/fimmu.2023.1134747)

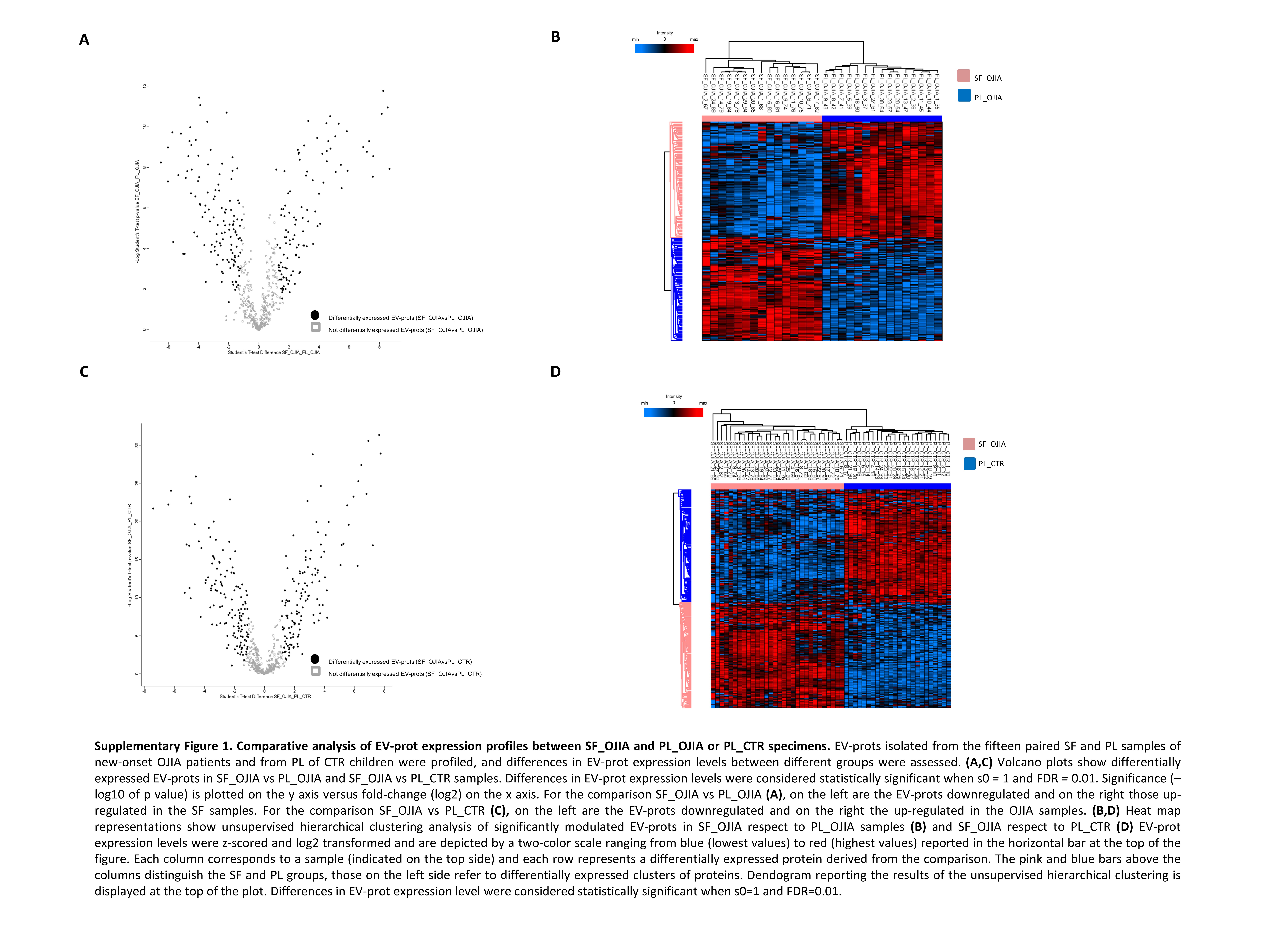

Supplement: Supplementary file 1 [file Image_1.jpg]
